# The Efficacy of Different Tenotomies in the Treatment of Lateral Epicondylitis: A Systematic Review

**DOI:** 10.3390/jcm13226764

**Published:** 2024-11-10

**Authors:** Ayub Ansari, Dania Shoaib, Yazan Tanbour, Charles R. Marchese, Benjamin J. Pautler, Abdullah Baghdadi, Sara Sloan, Jennifer F. Dennis

**Affiliations:** 1 College of Osteopathic Medicine, Kansas City University, Kansas City, MO 64106, USAyazan.tanbour@kansascity.edu (Y.T.); charles.marchese@kansascity.edu (C.R.M.); benjamin.pautler@kansascity.edu (B.J.P.); ssloan@kansascity.edu (S.S.); 2 College of Human Medicine, Michigan State University, Grand Rapids, MI 49503, USA; 3 Kansas College of Osteopathic Medicine, Kansas Health Science University, Wichita, KS 67202, USA

**Keywords:** lateral epicondylitis, tennis elbow, tenotomy, pain relief, VAS pain scale, MEPS questionnaire, DASH questionnaire, return to work

## Abstract

**Background:** Lateral epicondylitis impacts 1–3% of the population. It affects nearly half of all tennis players, primarily due to repetitive forearm muscle use leading to pain at the lateral elbow, particularly at the extensor carpi radialis brevis tendon. While conservative treatments resolve most cases, 4–11% of patients with persistent pain require surgery. Tenotomy is the gold standard for repair, but the research comparing the benefits of specific types of tenotomies (open, arthroscopic, percutaneous, ultrasonically assisted, and Tenex forms) is lacking. **Methods:** PubMed and Embase searches were conducted for articles focused on four tenotomy techniques. The inclusion criteria allowed for the use of randomized controlled trials (RCTs), prospective cohort studies, and comparative observational studies, while the exclusion criteria excluded meta-analyses. Following the PRISMA guidelines, the initial search resulted in 2327 articles. Once the inclusion and exclusion criteria were applied, 1702 articles underwent abstract screening. Finally, 232 articles proceeded to full-text screening, resulting in 37 articles undergoing data extraction. **Results:** The primary outcomes included functional improvement, pain relief, overall performance, and postoperative disability. The secondary outcomes included patient-reported satisfaction, return-to-work timeframes, and procedural complications. **Conclusions:** The tenotomy outcomes were similar, regardless of the method, indicating that discussions with patients about their specific outcome preferences may help guide tenotomy method selection.

## 1. Introduction

Lateral epicondylitis, commonly known as tennis elbow, is a musculoskeletal condition characterized by pain and tenderness located at the lateral elbow, specifically the lateral epicondyle [1]. It is primarily caused by repetitive use of the forearm muscles and tendons during activities that involve gripping and wrist extension, leading to muscular and tendinous microtears and degeneration [2]. The prevalence of lateral epicondylitis ranges from 1% to 3% in the general population, with higher rates observed amongst certain occupational groups [3,4], and nearly half of all tennis players [5]. The management of lateral epicondylitis includes various treatment approaches, including physical therapy, non-steroidal anti-inflammatory drugs (NSAIDs), corticosteroid injections, and surgical options [2,3,6]. The majority of cases of lateral epicondylitis respond well to conservative management, but roughly 4–11% of patients experience persistent pain despite the conservative treatment, eventually requiring surgical intervention [7]. Among the various available surgical interventions, tenotomy has gained attention as a potential treatment option.

Tenotomy involves the surgical division or cutting of the affected tendon to relieve tension, stimulate healing, and promote tissue regeneration, offering several potential benefits in treating lateral epicondylitis. It allows for the removal of degenerative tissue, reduces the load on the affected tendon, and promotes the healing process [8]. Additionally, tenotomy is considered a less invasive procedure compared to traditional open surgeries, resulting in smaller incisions, decreased postoperative pain, and faster recovery [3]. However, it is essential to consider the potential drawbacks of tenotomy, including the risk of iatrogenic damage to the surrounding structures, the potential for incomplete healing, and the need for proper rehabilitation to optimize outcomes [3,6].

Various tenotomy techniques, including the open, arthroscopic, percutaneous, and ultrasound-guided methods, have been developed to treat lateral epicondylitis [9,10,11,12,13]. A comprehensive evaluation of the existing evidence on the efficacy of various tenotomies is lacking. As such, the objective of this narrative review was to analyze the literature for the comparative efficacy of different tenotomy techniques for lateral epicondylitis concerning functional improvement, pain relief, and patient-reported outcomes. A review of this nature will be the first to compare all existing tenotomy options so researchers, providers, and patients alike are able to use the available data to guide their decisions. The comparative effectiveness of different tenotomy procedures, such as the open tenotomy, percutaneous needle tenotomy, arthroscopic tenotomy, and ultrasound-guided tenotomy methods, is discussed. This review aims to guide treatment decisions by comparing existing tenotomy options.

## 2. Materials and Methods

The following systematic review protocols were used in accordance with the Preferred Reporting Items for Systematic Review and Meta-Analysis (PRISMA) guidelines [14]. Registration was obtained through Prospero (CRD42023460324) on 10 November 2023. A thorough search was conducted on the PubMed and Embase databases. The relevant keywords included “lateral epicondylitis”, “tennis elbow”, “tenotomy”, “percutaneous needle tenotomy”, “arthroscopic tenotomy”, “functional improvement”, “recurrence rates”, and “long-term outcomes”. The Boolean operators “and” and “or” were used to further specify the search. The databases were searched from 15 August 2023 to 1 January 2024. Studies were then screened based on the inclusion and exclusion criteria outlined below.

### 2.1. Eligibility Criteria

Randomized controlled trials (RCTs), prospective cohort studies, and comparative observational studies (such as case-control studies) evaluating the efficacy of different tenotomy techniques for treating lateral epicondylitis in adults aged 18 years and older were included. Studies involving adults (aged 18 years and above) diagnosed with lateral epicondylitis (tennis elbow) were included. This review included studies assessing the use of various tenotomy techniques, including open tenotomy, percutaneous needle tenotomy, arthroscopic tenotomy, and endoscopic tenotomy, as the primary treatment modalities. Studies that included comparisons between different tenotomy techniques or comparisons against non-surgical interventions (such as conservative measures, physical therapy, or corticosteroid injections) were eligible. Studies reporting outcomes related to pain relief, functional improvement, patient-reported outcomes, complications, recurrence rates, and long-term outcomes were also included.

### 2.2. Exclusion Criteria

Studies were excluded if they were review articles, case reports, editorials, commentaries, or conference abstracts without original data. Non-English language studies and studies that did not report relevant outcome measures related to the efficacy of tenotomy techniques in treating lateral epicondylitis were also excluded.

### 2.3. Study Selection

The screening of the papers based on title, abstract, and full text was performed by four investigators (AA, DS, YT, CM). Any disagreements were resolved through discussion.

### 2.4. Data Extraction

Once the studies meeting the eligibility criteria were identified, data related to the primary and secondary outcomes were extracted. The primary outcomes included pain relief, functional improvement, and patient-reported outcomes. The secondary outcomes included complications, recurrence rates, return to work/sport, and long-term outcomes.

Pain relief was assessed based on the reduction in pain using the validated Visual Analog Scale (VAS). Functional improvement was evaluated by outcomes including grip strength and functional scores such as the Disabilities of the Arm, Shoulder, and Hand (DASH) questionnaire and the Mayo Elbow Performance Score (MEPS). Patient-reported outcomes were assessed in relation to the symptom satisfaction and overall improvement in the quality of life.

The complications evaluated included infection, nerve injury, tendon rupture, or issues with wound healing. The recurrence rate analysis included the symptom recurrence or the need for additional interventions during a defined follow-up period. The return to work/sport was assessed based on the time it took for the patients to return to these activities after tenotomy. Lastly, the long-term outcomes included the pain recurrence, functional outcomes, and patient satisfaction beyond the immediate post-treatment period.

### 2.5. Quality Assessment

While no formal quality assessment tools were employed, the methodological quality of the included studies was appraised based on factors such as study design, sample size, participant selection criteria, clarity in the reporting of interventions and outcomes, length of the follow-up, and potential sources of bias. Each study was independently assessed by two reviewers (AA and YT), and the discrepancies were resolved through consensus. This quality assessment informed the interpretation of the findings and the strength of the conclusions drawn.

### 2.6. Data Synthesis and Analysis

Given the expected heterogeneity across studies concerning the design, population characteristics, and outcome measures, a narrative synthesis approach was adopted. The studies were compared based on predefined criteria, including the study design, tenotomy technique used, sample size, patient demographics (age, gender), duration of symptoms, prior treatments, outcome measures reported, and duration of the follow-up. The synthesis focused on the data obtained from the randomized control trials (RCTs), prospective cohort studies, and comparative observational studies, all evaluating the efficacy of various tenotomy methods for lateral epicondylitis.

The differences in the outcomes across studies were handled by qualitatively summarizing the results, noting the consistencies and discrepancies among the studies. When studies reported similar outcomes using different measurement scales, efforts were made to standardize or convert the results to a common scale where feasible, facilitating comparison. In the cases where standardization was not possible, the findings were presented descriptively, and the impact of the different scales on the comparability was discussed. A meta-analysis was not conducted due to the anticipated variability in the study methodologies and the diverse nature of the tenotomy procedures.

The narrative synthesis comprehensively analyzed the associations between the different tenotomy techniques and the outcomes of interest, considering the collective evidence from the included studies. This involved grouping the studies by tenotomy technique and outcome measure, summarizing the findings within each group, and identifying the patterns, strengths, and limitations. This synthesis facilitated a nuanced understanding of the comparative efficacy of various tenotomy techniques for the treatment of lateral epicondylitis, providing valuable insights for clinical decision-making. Any outcomes with missing summary statistics were not reported in the tables here.

## 3. Results

**Figure 1:**
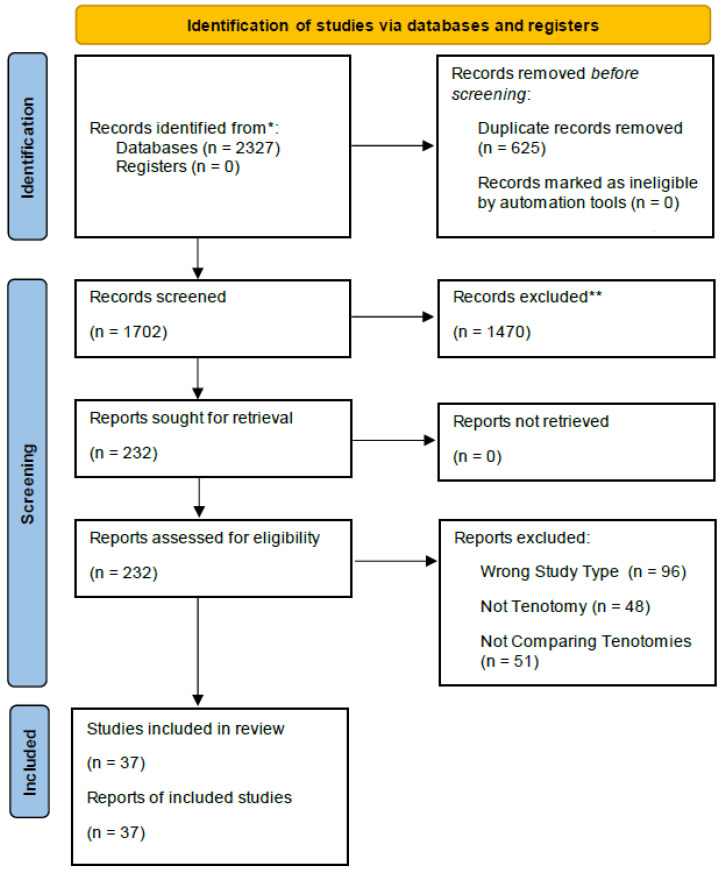
The PRISMA workflow diagram. * The number of records identified from the database and register searches. ** Any records excluded were excluded by a human; no automation tools were used.

The literature search yielded a total of 2327 articles, with the PubMed search yielding 1313 articles and the Embase search yielding 1014 articles. After the removal of duplicate articles (*n* = 625), 1702 articles underwent abstract screening. A total of 1470 articles were excluded, leaving 232 articles for full-text screening. During the full-text screening, 96 articles were excluded due to being the wrong study type, 48 articles were excluded due to not addressing a tenotomy procedure, and 51 articles were excluded for not comparing different methods of tenotomy. The removal of these 195 additional articles left a total of 37 articles eligible for data extraction (Figure 1).

The characteristics of the patients included in the studies reviewed are presented in Table 1. The study characteristics are reported in Table 2. The preoperative and postoperative study outcomes are reported in Table 3. The patient satisfaction and the return-to-work characteristics are presented in Table 4 and Table 5, respectively.

The preoperative and postoperative study outcomes are reported in Table 3. The patient satisfaction is presented in Table 4, and the return-to-work characteristics are presented in Table 5.

## 4. Discussion

### 4.1. Grip Strength Outcomes

Of the 37 studies included, 11 studies reported the grip strength after the utilization of different tenotomy methods. The grip strength was measured as either the total pounds or percentage change compared to preoperative levels. Eight studies utilized an arthroscopic procedure, with six of those showing an increase in grip strength postoperatively. Four arthroscopic procedures measured the overall grip power, with an average postoperative increase of 14.78 lbs, while the other two arthroscopic studies reported an increase in postoperative strength of 18.15%. The remaining two arthroscopic studies did not provide preoperative results. Two studies utilizing the open method recorded an increase in postoperative grip strength of 8.6 lbs. and 15%. These findings indicate that open procedures may offer reliable improvement in grip strength, with outcomes comparable to the arthroscopic method in some cases, especially when addressing more complex cases with direct access. Two studies utilized the ultrasound method and demonstrated an increase of 5.77 lbs. Only one study utilized a percutaneous method, which did not show any improvement between the preoperative and postoperative tests [51]. While the percutaneous approach did not yield improvement in grip strength, it may be best suited for cases where strength is not the primary focus, possibly favoring recovery time and reduced procedural impact. Notably, no studies measured the grip strength in the tenotomies performed using the Tenex procedure.

The weighted median values were not available due to the inconsistent reporting formats; however, we relied on the averages reported in the studies to summarize the data for comparison across techniques. Kim et al. [43] reported that while both the open and the arthroscopic procedures resulted in an increase in grip strength, the open procedure resulted in a higher change. Lee et al. reported a greater increase in grip strength with the ultrasonic approach compared to the arthroscopic surgical method [44]. Meknas and colleagues [45] showed an increase in strength using the open surgical method compared to the ultrasonic approach. Regarding grip strength, all methods, except for the percutaneous tenotomy, resulted in increased grip strength postoperatively. When the data from all studies were stratified and averaged based on the type of tenotomy, the arthroscopic method resulted in the largest increase in postoperative grip strength, both in the total weight (lbs) and the percentage of change. Interestingly, both Lee et al. [44] and Meknas et al. [45] compared the arthroscopic tenotomy to other methods (open and ultrasound) and reported higher levels of grip strength postoperatively in the other methods, rather than the arthroscopic method. This analysis reinforces that, while arthroscopic tenotomy generally results in increased postoperative grip strength, the open method and ultrasound-guided tenotomy may be similarly effective in some cases. Without the weighted median data, these reported averages provide comparative insight but limit definitive conclusions. These findings suggest that although the arthroscopic tenotomy may result in favorable outcomes, other methods like open and ultrasound-guided tenotomy can also be effective, depending on patient-specific factors.

### 4.2. VAS Pain Outcomes

Out of the 37 studies included in this review, 11 reported pain using the Visual Analog Scale (VAS), which utilizes a 10 cm line representing a continuum of pain with “no pain” being on the far left (0 cm) and “worst pain” being on the far right (10 cm). Patients mark on this line where they perceive their level of pain, and the distance from 0 cm is measured, with higher numbers signifying more pain [52]. Out of these eleven studies, three studies utilized the open surgical approach and reported a decrease in pain by an average of 4.6 on the VAS. The six studies that utilized the arthroscopic surgical method noted an average decrease by 4.43 on the VAS, and the three studies that utilized the percutaneous surgical method found an average decrease of 4.72 on the VAS. The two studies utilizing the ultrasound method indicated a decrease in pain by an average of 5.61. Boden et al. [40] reported a decrease in VAS score of 3.3 using the Tenex surgical method, which was slightly less than the other surgical techniques.

To ensure that the reductions in pain could be primarily attributed to the tenotomy type rather than confounding factors, the studies generally attempted to control variables such as the patient demographics (age, baseline pain level), co-existing conditions, and rehabilitation protocols post-surgery. Kim et al. [43] compared the open surgical method to the arthroscopic method and showed that the pain from the open surgical method decreased to a greater extent. Othman et al. [47] compared the arthroscopic method to the percutaneous method and showed a near-identical decrease in pain. Lee and colleagues [44] demonstrated a larger reduction in pain using the ultrasonic approach compared to the arthroscopic surgical method. Meknas et al. [45] noted an insignificant decrease in pain using the ultrasonic approach compared to the open surgical method. This consistency across the comparative studies reinforces the likelihood that the type of tenotomy, rather than other variables, contributed to the observed changes in pain levels.

### 4.3. Mayo Elbow Performance Score (MEPS) Outcomes

Ten studies reported patient function using the Mayo Elbow Performance Score (MEPS). The MEPS assigns points based on the categories of pain, motion, stability, and function for a maximum of 100 points, with higher scores signifying better outcomes [53]. Seven studies evaluated the MEPSs after arthroscopic tenotomy, with five of those seven studies indicating an average increase of 36.36 points. The remaining two studies did not provide preoperative MEPSs. Two studies utilized ultrasonic tenotomy and exhibited an increase of 43.4 points. A single study examined the MEPS after the open method, which showed a postoperative increase of 40 points, and a single study utilized percutaneous tenotomy, resulting in an increase of 18.5 points. No studies evaluated the MEPS after utilizing the Tenex procedure.

Meknas et al. [45] reported that both the open and ultrasonic groups achieved a full score (100 points) on the MEPS scale postoperatively. The ultrasonic group showed an improvement of 41.8 points, while the open group improved by 40 points compared to their preoperative scores. Lee et al. [44] demonstrated improvement in both the arthroscopic and ultrasound groups, with the ultrasonic group improving to a greater extent (41.8 points) compared to the arthroscopic group (40.2 points). All procedures—open, arthroscopic, percutaneous, and ultrasonic—resulted in increased MEPSs when comparing the preoperative and postoperative results. The ultrasonic method had the best performance with an average increase of 43.4 points, suggesting it may offer an advantage in improving elbow function. The open method also demonstrated significant functional improvement, indicating it could be advantageous for patients requiring substantial enhancement in elbow performance. While the arthroscopic method showed slightly lower improvements, its minimally invasive nature might be beneficial for patients prioritizing a quicker recovery with reasonable functional gains. The percutaneous method, although showing the least increase, may still be a viable option for patients preferring less invasive procedures with moderate functional improvement.

### 4.4. Disabilities of the Arm, Shoulder, and Hand (DASH) Outcomes

Fifteen studies evaluated patient dysfunction using the Disabilities of the Arm, Shoulder, and Hand questionnaire (DASH). Unlike the MEPS, which is a measure of ability, the DASH score is a measure of disability, assigning lower (decreasing) scores to less disabled individuals [54]. This questionnaire assesses the performance, severity, and impact of difficulty in social activities, work, sleep, and self-image caused by arm, shoulder, or hand disabilities [54]. Twelve studies evaluated the arthroscopic procedure using the DASH score. Eight arthroscopic studies provided both the preoperative and postoperative scores, showing an average decrease of 32.84 points. The remaining four arthroscopic studies did not provide preoperative scores, so comparisons could not be made. Three studies evaluated the DASH score after percutaneous tenotomy, with an average decrease of 32.33 points. Two studies evaluated open tenotomy and demonstrated a decrease of 45.15 points. A single study evaluated the DASH score after the Tenex procedure, which decreased by a total of 23.4 points. No studies utilizing the ultrasound procedure were evaluated using the DASH score.

Kim et al. [43] indicated a decrease in both the open and arthroscopic surgical methods, with the open method resulting in a larger reduction. Soheim et al. [49] also indicated a decrease in both the open and arthroscopic surgical methods; however, the two methods showed a nearly equivalent decrease in DASH scores. Although we lack the weighted median values to offer a more refined comparison, the open tenotomy appears to result in the most significant reduction in DASH scores on average, suggesting potentially greater improvement in function and reduced disability postoperatively. This suggests that the open method may offer advantages in reducing disability and improving the overall arm function. The arthroscopic and percutaneous methods also demonstrated significant decreases in DASH scores, indicating they are effective options, particularly for patients seeking less invasive procedures with considerable functional benefits. The Tenex procedure showed the smallest decrease, which may be suitable for patients seeking moderate improvement with minimally invasive intervention.

### 4.5. Patient Satisfaction Outcomes

The patient satisfaction following tenotomy was reported in 15 studies, and was described as very satisfied, satisfied, or dissatisfied. The studies exploring the open tenotomy procedure reported that 76.67% were very satisfied with the results. No studies investigating open tenotomy supplied data displaying any patients recording “satisfied” or “dissatisfied” responses. The studies that investigated the arthroscopic procedure reported that 78.14% of patients were very satisfied, 75.25% of patients were satisfied, and 7.14% of patients were dissatisfied. The studies investigating percutaneous tenotomy reported that 36.84% of patients were very satisfied, 65.47% of patients were satisfied, and 10.52% of patients were dissatisfied. A single study investigating the Tenex procedure reported that 80% of patients were satisfied. These results suggest that the Tenex procedure resulted in the highest number of patients reporting being very satisfied. On the other hand, the percutaneous method resulted in the smallest number of patients reporting being very satisfied, and the highest amount of patients reporting being dissatisfied with their results.

These results suggest that the Tenex procedure resulted in the highest percentage of patients reporting being very satisfied, indicating it may have advantages in terms of patient satisfaction, potentially due to its minimally invasive nature and quicker recovery times. The open and arthroscopic methods also had high satisfaction rates, showing they are generally well received by patients, possibly due to their effectiveness in improving symptoms and function. The percutaneous method resulted in the lowest percentage of patients reporting being very satisfied and the highest percentage of dissatisfaction, suggesting it may be less favorable in terms of patient satisfaction, possibly due to less optimal outcomes or expectations not being met. The levels of satisfaction are well reported across the literature. However, it is unclear whether the satisfaction was measured immediately after surgery or after a prolonged period, which is a limitation of this review.

### 4.6. Return-to-Work Outcomes

Eight studies reported data on the return-to-work timeframes. The return to work after open tenotomy was recorded in two studies, with an average timeframe of 4.9 weeks. The data after arthroscopic procedures were reported in seven studies, with an average return-to-work time of 8.76 weeks. One single study evaluated the return-to-work time after utilizing the percutaneous technique at three weeks. Though the average return-to-work time was shorter when comparing open to arthroscopic tenotomies, Kim et al. [43] indicated that the patients that received arthroscopic microtenotomies returned to work sooner than those that received the open method. Globally, the percutaneous method resulted in the lowest return-to-work timeframe, but Othman [47] reports that the percutaneous method and arthroscopic method return-to-work times were identical at around three weeks. Based on the available data, the average return-to-work time was lowest after the percutaneous procedure, but this generalization is hindered by the small sample size (*n* = 1 study). These findings suggest that the percutaneous method may offer advantages in facilitating a quicker return to work, which could be particularly beneficial for patients needing minimal downtime. The open method also showed a relatively short return-to-work timeframe, indicating it may be advantageous for patients requiring substantial functional improvement without a prolonged recovery. While the arthroscopic method shows a slightly longer average return-to-work time, it may still be favorable due to its minimally invasive nature, balanced recovery time, and positive functional outcomes.

### 4.7. Complication Outcomes

Complications were reported by seven studies. Ankem et al. [15] reported synovial fistulae in eight patients that resolved spontaneously, portal superficial infections in two patients, and a mild anterior capsular contracture in one patient using the arthroscopic method. Babaqi et al. [17] reported one patient with radial nerve palsy and one with a superficial wound infection using the arthroscopic method. Kim et al. [43] reported a superficial infection in the open surgery group but none in the arthroscopic group. Lee et al. [44] reported that one patient using the arthroscopic technique needed a revision surgery, and one patient who received an ultrasound-based microtenotomy required a revision surgery after a rupture of the proximal edge of the ECRB. Miyazaki and colleagues [45] reported a single patient who received an arthroscopic tenotomy developed reflexive sympathetic dystrophy on the operated arm. Martinez et al. [21] reported complications in 28.5% of cases, including altered sensitivity, extension deficit, synovial plica, and synovitis, using the arthroscopic method. Soeur and colleagues [27] utilized the arthroscopic method and reported complications in four patients, including the subjective sensation of elbow instability and persistent pain, while two patients required further surgery.

This study was not without limitations. Very few of the studies included within this review directly compared two or more tenotomy methods. While the measurement parameters were consistent, the implementation of these measurement tools by the various authors might have differed, making comparison difficult. The stratification of data by the various tenotomy methods further lowered the sample size. Another limitation for this study involves the potential for missed literature. While the initial literature search was expansive, the literature within other databases or literature in different languages may exist. Future studies further comparing these different tenotomy methods using the measurement tools implemented by the same individuals are needed, and would allow for a more accurate comparison.

## 5. Conclusions

This systematic review evaluated the efficacy of different tenotomy techniques for treating lateral epicondylitis. The findings suggest that the arthroscopic method generally results in the largest increase in postoperative grip strength, making it a strong option for patients seeking significant strength improvement. Ultrasound-guided tenotomy demonstrated the greatest reduction in pain, indicating its potential advantage for patients prioritizing pain relief. Open tenotomy showed significant improvements in both grip strength and reduction of disability as measured by DASH scores, and may be particularly beneficial in complex cases requiring direct tendon access. The percutaneous method facilitated the quickest return to work, which could be advantageous for patients needing minimal downtime, despite showing less improvement in grip strength. the patient satisfaction was high across most techniques, with the Tenex procedure reporting the highest satisfaction rates. The complication rates varied among the methods, but were generally low. In conclusion, each tenotomy technique offers specific advantages, and the choice of procedure should be tailored to individual patient needs and priorities. Further high-quality, comparative studies are necessary to provide more definitive guidance on optimizing the treatment outcomes for lateral epicondylitis.

## Figures and Tables

**Table 1:** Characteristics of patients undergoing tenotomy procedures of included studies.

Study	Surgical Technique	Number of Elbows, Total, Individual Treatment Group (Treatment)	Age, Years, Mean ± SD (Min − Max)	Sex (*n*, %)	Symptom Duration, Months, Mean ± SD (Min − Max)	Duration of Nonoperative Treatment, Months, Mean ± SD (Min − Max)	Dominant Hand Affected (%)	Mean Follow-Up, Months, Mean ± SD (Min − Max)
Ankem et al. [15]	A	102	NA	NA	NR	≥6	NR	36 (24–45)
Arrigoni et al. [16]	A	18	46 (25–59)	M (5, 27.7%); F (13, 72.3%)	NR	≥12	55%	24 (24–30)
Babaqi et al. [17]	A	33	33.7 (24–42)	M (22, 66.7%); F (11, 33.3%)	NR	16.3 (6–36)	55%	14.4 (12–24)
Baraza et al. [18]	A	26	48 (27–57)	NA	NR	≥6	NR	72 (12–132)
Behazin et al. [19]	A	11	42 ± 6.8	M (3, 27%); F (8, 73%)	18	NR	90%	12
Das et al. [20]	A	125	M, 47 F, 45	M (40, 36%); F (71, 64%)	NR	NR	NR	52.8 (≥24)
Martynetz et al. [21]	A	15	46 (23–56)	M (8, 57%); F (6, 43%)	NR	30 (18–72)	80%	41 (24–72)
Matache et al. [22]	A	68	NA	NA	≥6	3	NR	24
Miyazaki et al. [23]	A	20	41.7	M (8, 40%); F (12, 60%)	28.5 (6–136)	≥6	65%	20 (12–48)
Oki et al. [24]	A	23	49	M (5, 21.7%); F (18, 78.3%)	32 (6–338)	≥6	NR	24
Saremi et al. [25]	A	40	42.9 ± 6.4	M (12, 30%); F (28, 70%)	10 (6–18)	≥6	55%	42
Shim et al. [26]	A	53	50 (27–77)	M (23, 43.4%); F (30, 56.6%)	NR	>12	58%	30 (24–49)
Soeur et al. [27]	A	35	48 ± 8.4	M (20, 57%); F (15, 43%)	18 (6–106)	≥6	NR	48 (12–144)
Torudom et al. [28]	A	22	44.9 ± 8.5	M (8, 46.3%); F (14, 53.7%)	20.6 ± 5.5	18.3 ± 3.3	NR	NR
Vander Voort et al. [29]	A	13	NA	NA	NR	6	NR	12
Verhaar et al. [30]	A	63	45 (25–67)	M (42, 66.7%); F (21, 33.3%)	12	0.96 (0.3–48)	84%	59 (50–65)
Amroodi et al. [31]	O1	24	38.5 (25–64)	M (9, 37.5%); F (15, 62.5%)	44.4	≥12	62.5%	34.8
Lungu et al. [32]	O1	64	NA	NA	≥6	≥6	NR	12
Solheim et al. 2011 [33]	O1	80	46 (34–64)	M (38, 49%); F (39, 51%)	13 (6–72)	≥6	71%	48
Thomas et al. [34]	O1	24	(38–59)	M (10, 55.5%); F (8, 44.6%)	23	NR	58%	NR
Carlier et al. [35]	P	261	47.6 ± 8.3	M (127, 50.4%); F (125, 49.6%)	28.9 ± 24.4	≥6	NR	3
Kaleli et al. [36]	P	26	NA	M (12, 46%); F (14, 54%)	8.9	Conservative methods before surgery	NR	32
Nazar et al. [37]	P	30	55 (26–71)	M (7, 29%); F (17, 71%)	40 (7–120)	≥2	77%	36 (1–71)
Yigit et al. [38]	P	47	46 (28–66)	M (19, 46.3%); F (22, 53.7%)	NR	>6	74%	NR
Bhandari et al. [39]	A, O1	72, 30 (A), 42 (O1)	NA	M (32, 44%); F (40, 56%)	9	9	NR	≥3
Boden et al. [40]	R, T	62, 32 (R), 30 (T)	47 ± 12 (R) 51 ± 8 (T)	M (22, 69%) (R), F (10, 31%) (R) M (18, 60%) (T), F (12, 40%) (T)	26 ± 24 (R) 25 ± 21 (T)	NR	NR	17 (R) 10 (T)
Choudhury et al. [41]	A, C	47, 24 (A), 23 (C)	NA	NA	NR	NR	NR	≥42
Dabkara et al. [42]	C, A	47, 23 (C), 24 (A)	NA	NA	NR	>6	NR	≥42
Kim et al. [43]	O2, A	68, 34 (O2), 34 (A)	48 ± 8.1 (O2) 49 ± 7.8 (A)	O2 [M (16, 47%); F (18, 53%)] A [M (12, 35%); F (22, 65%)]	25.4 ± 6.1 (O2) 26.1 ± 6.8 (A)	NR	76% (O2) 71% (A)	NR
Lee et al. [44]	A, U	46, 24 (A), 22 (U)	51.25 ± 8.57 (A) 51.59 ± 5.75 (U)	A [M (11, 46%); F (13, 34%)] U [ M (8) (36%); F (14, 64%)]	26.17 ± 8.14 (A) 23.91 ± 6.98 (U)	≥6	63% (A) 68% (U)	24
Meknas et al. [45]	O1, U	24, 11 (O1), 13 (U)	49.2 (36–62) (O1) 46.2 (30–64) (U)	M (13, 54%); F (11, 46%)	28 (12–60) (O1) 22 (12–50) (U)	≥12	NR	75.5 (O1) 68.4 (U)
Merolla et al. [46]	A, R	101, 50 (A), 51 (R)	all >18	NA	0.56 (A) 0.44 (R)	≥4	NR	24
Othman et al. [47]	A, P	33, 14 (A), 19 (P)	42 48	A [M (8, 57%); F (6, 43%)] P [M (12, 63%); F (7, 37%)]	>6	>6	NR	12 (A) 10 (P)
Radwan et al. [48]	U, P	56 29 (U), 27 (P)	40.14 (U) 39.26 (P)	U [M (15, 52%); F (14, 48%)] P [M (18, 67%); F (9, 33%)]	16.72 (U) 18.26 (P)	≥6	66% (U) 63% (P)	12
Solheim et al. 2013 [49]	A, O1	305 225 (A), 80 (O1)	46 ± 8	~50% females in both groups	24 ± 23 (A) 19 ± 15 (O1)	≥6	71% (A) 76% (O1)	48
Watts et al. [50]	O2, R	81 41 (O2), 40 (R)	NA	NA	≥6	≥4	NR	≥12
Degen et al. [51]	NA	3863	71.6% aged <65 years	M (1654, 42.8%); F (2209, 57.2%)	NR	NR	NR	≥24

A: arthroscopic; C: continued intensive conservative management; D: drilling; NA: not applicable; NR: not reported; O1: open lateral extensor release; O2: open Nirschl; P: percutaneous release; R: platelet-rich plasma; T: Tenex; U: ultrasound-based microtenotomy.

**Table 2:** Study characteristics of reported tenotomy procedures.

Study	Patient Outcome Measurements
Amroodi et al. [31]	VAS
Ankem et al. [15]	Grip Strength, MEPS, DASH
Arrigoni et al. [16]	MEPS, DASH, Andrews–Carson Score
Babaqi et al. [17]	MEPS, VAS, DASH, PRTEE
Baraza et al. [18]	MEPS
Behazin et al. [19]	MEPS, DASH, PRTEE
Bhandari et al. [39]	NA
Boden et al. [40]	VAS, DASH, EuroQol-5D (EQ5D) Scores
Carlier et al. [35]	Grip Strength, MEPS, VAS, DASH, PRTEE, Elbow Self-Assessment Score (ESAS)
Choudhury et al. [41]	NA
Dabkara et al. [42]	NA
Das et al. [20]	NA
Degen et al. [51]	Incidence of Failure/Revision Surgery, Time to Revision Surgery
Kaleli et al. [36]	Pain Relief
Kim et al. [43]	Grip Strength, VAS, DASH
Lee et al. [44]	Grip Strength, MEPS, VAS, DASH, Flexion–Extension Arc
Lungu et al. [32]	NA
Martynetz et al. [21]	MEPS, DASH
Matache et al. [22]	NA
Meknas et al. [45]	Grip Strength, MEPS, VAS
Merolla et al. [46]	Grip Strength, VAS, PRTEE
Miyazaki et al. [23]	AMA Criteria
Nazar et al. [37]	NA
Oki et al. [24]	Grip Strength, VAS, DASH, JOA score
Othman [47]	VAS, DASH
Radwan et al. [48]	NA
Saremi et al. [25]	Grip Strength, VAS, DASH
Shim et al. [26]	Grip Strength, DASH, Nirschl Score
Soeur et al. [27]	DASH
Solheim et al. 2011 [33]	DASH
Solheim et al. 2013 [49]	DASH
Thomas et al. [34]	Excellent Pain Relief
Torudom et al. [28]	Grip Strength, VAS
Vander Voort et al. [29]	DASH, Single Assessment Numerical Evaluation (SANE) Scores
Verhaar et al. [30]	Grip Strength, Pain, and Tenderness
Watts et al. [50]	Improvement in Pain
Yigit [38]	MEPS, Postoperative Pain Score

DASH: Disabilities of the Arm, Shoulder, and Hand questionnaire; JOA: mean Japanese Orthopedic Association score; MEPS: Mayo Elbow Performance Score; PRTEE: Patient-Rated Tennis Elbow Evaluation; VAS: Visual Analog Scale.

**Table 3:** Preoperative and postoperative study measures of different outcome instruments reported.

	Open	Arthroscopic	Percutaneous	Ultrasonic	Tenex
**Grip strength** [**Pre**; **Post** (**Difference**)]					
Ankem et al. [15]		NR; >35% (−)			
Carlier et al. [35]			8.3 lbs; 8.3 ± 10 lbs (0)		
Kim et al. [43]	79.4% ± 3.5%; 94.4% ± 4.1% (+15)	77.3% ± 3.3%; 91% ± 3.7% (+13.7%)			
Lee et al. [44]		20.2 ± 6.35 lbs; 25.32 ± 6.55 lbs (+5.12)		19.97 ± 6.74 lbs; 26.00 ± 6.91 lbs (+6.03)	
Meknas et al. [45]	29.1 ± 12.9 (15–54) lbs; 37.7 ± 6.1(28–42) lbs (+8.6)			28.3 ± 16.9 (8–54) lbs; 33.8 ± 13.1 (8–58) lbs (+5.5)	
Merolla et al. [46]		26.6 ± 5.6 lbs; 47.3 ± 4.8 lbs (+20.7)			
Oki et al. [24]		66.1%; 88.7% (+22.6)			
Saremi et al. [25]		NR; 38.65 ± 19.16 lbs (−)			
Shim et al. [26]		NR; 4.3% ± 30.3% (−)			
Torudom et al. [28]		18.6 ± 3.1 lbs; 35.3 ± 3.8 lbs (16.7)			
Verhaar et al. [30]		18.8 ± 11.5 lbs; 35.4 ± 13.6 lbs (+16.6)			
**Mayo Elbow Performance Score**, **MEPS** [**Pre**; **Post** (**Difference**)]					
Ankem et al. [15]		57; 89 (+32)			
Arrigoni et al. [16]		NR; 82.5 (range, 60–100) (−)			
Babaqi et al. [17]		61.82; 94.10 (+32.9)			
Baraza et al. [18]		47.5; 90.2 (+42.7)			
Behazin et al. [19]		56 ± 9; 90 ± 10 (+34)			
Carlier et al. [35]			67.4; 85.9 (+18.5)		
Lee et al. [44]		55.2 ± 6.3; 95.4 ± 8.7 (+40.2)		53.9 ± 6.7; 95.7 ± 6.8 (+41.8)	
Martynetz et al. [21]		NR; 90 (−)			
Meknas et al. [45]	60 (30–85); 100 (70–100) (+40)			55 (40–80); 100 (65–100) (+45)	
Yigit [38]			NR; 82 (40–100) (−)		
**Visual Analog Pain Scale** (**VAS**) [**Pre**; **Post** (**Difference**)]					
Amroodi et al. [31]	7.2; 3.5 (−3.7)				
Babaqi et al. [17]		8.64; 1.48 (−7.16)			
Boden et al. [40]					5.5 ± 0.8; 2.2 ± 0.5 (−3.3)
Carlier et al. [35]			7.4 ± 1.14; 4 ± 2.2 (−3.4)		
Kim et al. [43]	5.8 ± 0.9; 0.8 ± 0.7 (−5)	1.2 ± 0.9; 0.8 ± 0.7 (−0.4)			
Lee et al. [44]		7.33 ± 1.05; 3.27 ± 1.07 (−4.06)		7.27 ± 0.94; 1.75 ± 1.22 (−5.52)	
Meknas et al. [45]	6.4 ± 1.5 (4–8); 1.3 ± 1.7 (0–5) (−5.1)			7.1 ± 1.6 (5–10); 1.4 ± 2.3 (0–5) (−5.7)	
Merolla et al. [46]		9 (8–10); 5 (3–6) (−4)			
Othman [47]		9.1; 2 (−7.1)	9; 2.1 (−6.9)		
Saremi et al. [25]		7.05; 3.2 (−3.85)			
Torudom et al. [28]		6.7; NR (−)			
**Disabilities of the Arm**, **Shoulder**, **and Hand Questionnaire** (**DASH**) [**Pre**; **Post** (**Difference**)]					
Arrigoni et al. [16]		NR; 20.14 (range, 5–57.5) (−)			
Babaqi et al. [17]		55.53; 10.39 (−44.94)			
Behazin et al. [19]		56 ± 15; 15 ± 16 (−41)			
Boden et al. [40]					35.9 ± 5.0; 12.5 ± 3.4 (−23.4)
Carlier et al. [35]			56.1; 23.1 (−33)		
Kim et al. [43]	70.7 ± 15.1; 29.3 ± 18.4 (−41.4)	69.2 ± 16.4; 40.4 ± 16.2 (−28.8)			
Martynetz et al. [21]		NR; 57 (−)			
Oki et al. [24]		32; 15 (−17)			
Othman [47]		72; 48 (−24)	70; 50 (20)		
Saremi et al. [25]		63.18; 25.68 (−37.5)			
Shim et al. [26]		NR; 15.9 ± 19.1 (−)			
Soeur et al. [27]		NR; 17.1 ± 24.2 (−)			
Solheim et al. 2011 [33]			61 ± 16; 17 ± 20 (−44)		
Solheim et al. 2013 [49]	60.5 ± 16.5; 11.6 ± 15.6 (−48.9)	60.2 ± 15.4; 17.8 ± 19.4 (−42.4)			
Vander Voort et al. [29]		54.0; 26.9 (−27.1)			

The Disabilities of the Arm, Shoulder, and Hand Questionnaire (DASH) is the perceived decreased ability caused by arm, shoulder, and hand disabilities; the grip strength is reported as the total pounds (lbs) or percentage; the Mayo Elbow Performance Score (MEPS) has a maximum score of 100 points; the Visual Analog Pain Scale (VAS) reports the perceived pain values using a scale of 0, “no pain”, through 10, “worst pain”. The differences in the pre- and post-measurements are calculated. Positive changes in grip strength and MEPS denote improved patient outcome(s); negative change values for the DASH and VAS are associated with perceived improvement.

**Table 4:** Patient satisfaction scores.

Patient Satisfaction	Open	Arthroscopic	Percutaneous	Tenex
Very Pleased				
Amroodi et al. [31]	O1	24	38.5, 25–64	NA
Kim et al. [43]				
Oki et al. [24]				
Othman et al. [47]				
Solheim et al. [33]	O1	80	46, 34–64	23
Solheim et al. [49]	A, O1	225, 80	46	Patients with TE refractory to conservative care for at least 6 months
Satisfied				
Babaqi et al. [17]		93.5%		
Boden et al. [40]				80%
Carlier et al. [35]			78.3% ± 19.4	
Das et al. [20]		73%		
Martynetz et al. [21]		85%		
Miyazaki et al. [23]		65%		
Othman [47]		42.85%	52.63%	
Vander Voort et al. [29]		92.3%		
Dissatisfied				
Othman [47]		7.14%	10.52%	

A: arthroscopic; NA: not applicable; O1: open lateral extensor release.

**Table 5:** Patient return-to-work timeframes.

	Open (Range)	Arthroscopic (Range)	Percutaneous
Amroodi et al. [31]	4.8 weeks (2–9 weeks)		
Babaqi et al. [17]		8 days (3–21 days)	
Choudhury et al. [41]		4.64 mo	
Dabkara et al. [42]		6.13 mo	
Kim et al. [43]	5 weeks (3–7 weeks)	3 weeks (1–6 weeks)	
Oki et al. [24]		8.6 weeks	
Othman [47]		3 weeks	3 weeks
Saremi et al. [25]		18 days	

## Data Availability

The original contributions presented in the study are included in the article.
